# Myocyte contractility can be maintained by storing cells with the myosin ATPase inhibitor 2,3 butanedione monoxime

**DOI:** 10.14814/phy2.12445

**Published:** 2015-06-28

**Authors:** Charles S Chung, Charles Mechas, Kenneth S Campbell

**Affiliations:** Department of Physiology and Center for Muscle Biology, University of KentuckyLexington, Kentucky

**Keywords:** BDM (2,3-Butanedione monoxime), blebbistatin, calcium, cardiomyocyte, cross-bridge, sarcomere

## Abstract

Isolated intact myocytes can be used to investigate contractile mechanisms and to screen new therapeutic compounds. These experiments typically require euthanizing an animal and isolating fresh cells each day or analyzing cultured myocytes, which quickly lose their rod-shaped morphology. Recent data suggest that the viability of canine myocytes can be prolonged using low temperature and N-benzyl-p-toluene sulfonamide (an inhibitor of skeletal myosin ATPase). We performed similar studies in rat myocytes in order to test whether the cardiac myosin ATPase inhibitors 2,3-Butanedione monoxime (BDM) and blebbistatin help to maintain cell-level function over multiple days. Myocytes were isolated from rats and separated into batches that were stored at 4°C in a HEPES-buffered solution that contained 0.5 mmol L^−1^ Ca^2+^ and (1) no myosin ATPase inhibitors; (2) 10 mmol L^−1^ BDM; or (3) 3 *μ*mol L^−1^ blebbistatin. Functional viability of myocytes was assessed up to 3 days after the isolation by measuring calcium transients and unloaded shortening profiles induced by electrical stimuli in inhibitor-free Tyrode's solution. Cells stored without myosin ATPase inhibitors had altered morphology (fewer rod-shaped cells, shorter diastolic sarcomere lengths, and membrane blebbing) and were not viable for contractile assays after 24 h. Cells stored in BDM maintained morphology and contractile function for 48 h. Storage in blebbistatin maintained cell morphology for 72 h but inhibited contractility. These data show that storing cells with myosin ATPase inhibitors can extend the viability of myocytes that will be used for functional assays. This may help to refine and reduce the use of animals in experiments.

## Introduction

Intact myocytes are frequently used to investigate the molecular mechanisms that underlie contraction and to screen potential therapies for cardiac disease (King et al. 2011; Malik and Morgan 2011; Aronson and Krum 2012; Campbell et al. 2013; Chung and Campbell 2013). Many experiments can be performed using myocytes that have been cultured but these cells typically exhibit altered morphology (e.g., nonrectangular shape and misaligned sarcomeres) that make it difficult to assess contractility. Thus groups studying how mutations and/or drugs alter calcium transients and cell shortening often isolate fresh cells on a nearly daily basis.

Isolation procedures require euthanizing an animal but often yield millions of cells. Assays of contractile properties can be time-consuming so many groups will study only a few tens of cells on the day of the isolation. The remaining cells are unlikely to be functional the following day and are commonly discarded. If the left-over cells could be preserved and studied for several days in a row, researchers could reduce the number of animals that need to be euthanized for the experiments. The researchers would also save time and resources. A method that prolongs the viability of myocytes for use in functional assays could thus accelerate research relating to cardiac disease.

Recently, Abi-Gerges et al. (2013) reported that the morphology and shortening profiles of canine myocytes could be maintained for at least 1 week by storing them at low temperature in a solution containing N-benzyl-p-toluene sulfonamide (BTS). This compound was previously shown to inhibit the ATPase of skeletal muscle myosin (Cheung et al. 2002).

We performed experiments to test whether a similar approach can preserve the morphology and prolong the contractile viability of rat myocytes which have faster maximally activated ATPase rate than canine cells. Instead of BTS, we chose to test 2,3-butanedione 2-monoxime (BDM) and blebbistatin, because these compounds are already used by many investigators to inhibit myosin ATPase in rodent cardiac cells. BDM is a myosin II ATPase inhibitor (Louch et al. 2011), which can exhibit phosphatase activity (Wiggins et al. 1980). It is commonly used to inhibit contractility during isolation of myocytes, which is thought to improve cell yield (Peeters et al. 1995; Louch et al. 2011). Blebbistatin is an ATPase inhibitor that reduces the actin-binding affinity of myosin (Kovács et al. 2004). It has been used to uncouple contractility from electrical stimulation and calcium release (Farman et al. 2008) and to investigate passive cardiac mechanics (King et al. 2011).

## Materials and Methods

### Animals

Eight female Sprague–Dawley rats (7 ± 1 months of age, Charles River Laboratories) were used in this study. All animal use was approved by the Institutional Animal Use and Care Committee of the University of Kentucky.

### Myocyte isolation

Myocytes were isolated as previously described (Louch et al. 2011; Campbell et al. 2013; Chung and Campbell 2013). Briefly, rats were heparinized (700 U IP), anesthetized (Sodium Pentobarbital 50 mg·kg^−1^ IP), and killed by exsanguination. Excised hearts were immediately placed in a cold (4°C), oxygenated (>10 ppm) Perfusion Solution (PS; in mmol L^−1^: 113 NaCl, 4.7 KCl, 0.6 KH2PO4, 1.2 MgSO4, 12 NaHCO3, 10 KHCO3, 10 HEPES, 30 Taurine, 5.5 glucose) with 10 mmol L^−1^ BDM. The aortas were then cannulated so that the hearts could be flushed until they were clear of blood. This procedure was started using 5 mL of the cold PS plus 10 mmol L^−1^ BDM and finished with 5 mL of warm PS plus 10 mmol L^−1^ BDM (37°C, via gravity perfusion at 90 mmHg pressure).

The hearts were then perfused with digestion solution (PS with 2 *μ*mol L^−1^ Ca^2+^ and 4.8 mg of Liberase TH™ (Roche, Indianapolis, IN), with 10 mmol L^−1^ BDM) for ∼10 min before being removed from the cannula. The tissue was then minced using small ceramic-coated scissors, lightly agitated to dissociate cells, and then resuspended in an enzyme stopping solution (PS with 10% fetal bovine serum and 12 *μ*mol L^−1^ Ca^2+^) for 5 min. Cells were allowed to settle by gravity into a pellet at the bottom of the vial. The supernatant was then drawn off and replaced with a second enzyme stopping solution (perfusion solution with 5% fetal bovine serum and 12 *μ*mol L^−1^ Ca^2+^). Extracellular Ca^2+^ was then raised to 0.5 mmol L^−1^ using a four-step calcium ladder. The products of this isolation were then divided between at least 24 Eppendorf tubes. Each tube contained 25–75 *μ*L of cells in a total volume of 500 *μ*L. All solutions were pH = 7.3 at room temperature.

### Myocyte storage

Tubes that would be stored were allowed to settle and their supernatant was replaced by one of three storage solutions: (1) 500 *μ*L PS with 0.5 mmol L^−1^ Ca^2+^, (PS group) 2) 500 *μ*L PS with 0.5 mmol L^−1^ Ca^2+^, and 10 mmol L^−1^ BDM (PS + BDM group), or 3) 500 *μ*L PS with 0.5 mmol L^−1^ Ca^2+^and 3 *μ*mol L^−1^ blebbistatin (PS + Bleb) group. The tubes were stored at 4°C. No solution changes were performed during storage. BDM was acquired from Sigma Aldrich (B0753) and blebbistatin was acquired from CalbioChem (203390).

### Myocyte morphology

Cell morphology was assessed by resuspending cells from a randomly selected Eppendorf tube and pipetting a droplet onto a microscope slide. Cells were then imaged using a standard laboratory microscope. Cell shape, presence of membrane blebbing, and clarity of the striation pattern were assessed qualitatively. The proportion of rod-shaped cells (i.e.,, those cells with a length/width ratio of at least 2.5) was quantified and compared to the proportion that was measured on the day that the cells were isolated. Approximately 300 cell-like bodies were counted in each condition (minimum 88, maximum 884 cells per condition) from four isolations.

### Myocyte function

Samples were prepared for functional measurements by allowing the myocytes to settle and replacing the supernatant with fresh PS containing 0.5 mmol L^−1^ Ca^2+^ but no myosin ATPase inhibitors. This was repeated three times with 20 min in between to remove as much of the initial storage medium as possible. After the final solution change, extracellular Ca^2+^ was raised to 1 mmol L^−1^ and the cells were allowed to acclimate for at least 1 h.

Functional measurements were performed in an oxygenated (>10 ppm) Tyrode's solution (in mmol L^−1^: 140 NaCl, 5.4 KCl, 1.8 CaCl2, 1 MgCl2, 10 HEPES, 10 glucose, pH 7.3) as previously described (Campbell et al. 2013; Chung and Campbell 2013). Briefly, 40 *μ*L of a solution containing calcium tolerant cells was added to 230 *μ*L of Fura loading solution (Tyrode's solution with 0.02% pluronic acid and 2 *μ*mol L^−1^ of the cell permeable fluorescent calcium indicator Fura-2AM (Life Technologies, Grand Island, NY)). Loading solutions were mixed fresh on each experimental day and allowed to incorporate into the cytosol for 15 min before assays were performed. Loading was ended by transferring 30 *μ*L of cells into 150 *μ*L of fresh Tyrode's solution. After a further 15 min, these cells were transferred to an experimental chamber that was continuously perfused with oxygenated Tyrode's solution. Temperature was controlled at 25°C using an in-line heater (HPRE2, Cell MicroControls, Norfolk, VA) and a custom 1.5 watt heating source formed from power resistors embedded around the chamber. After settling to the cover glass, cells were continuously paced at 0.5 Hz using a 5 msec duration bipolar excitation.

Measurements were performed using apparatus that has been described previously (Campbell et al. 2013; Chung and Campbell 2013). Briefly, sarcomere length shortening profiles were measured using a high speed video camera and converted to an analog voltage signal using a D/A converter (901A HVSL and 903A, Aurora Scientific, Aurora, Ontario Canada). Calcium transients were measured by monitoring Fura-2 fluorescence at 510 nm in response to dual wavelength excitation at 340 and 380 nm (Ratiomaster DeltaRAM X illuminator and D-104 Photometer, Photon Technology International, Birmingham, NY). Excitation switching, temperature, and pacing were computer controlled using a customized program written in MATLAB (Mathworks, Natick, MA) and 16 bit data acquisition cards (DAP5216a, Microstar Laboratories, Bellevue, WA) operating at 2 kHz.

**Figure 1:**
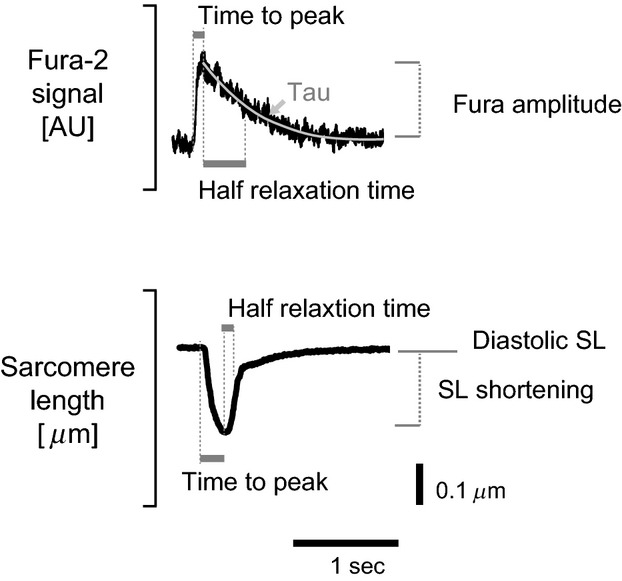
Parameters reported in this study.

Calcium transients and unloaded shortening profiles were analyzed offline using custom-written software developed in MATLAB. Calcium fluorescence ratios were calculated by dividing the emission (510 nm) intensity values recorded during successive excitations by 340 nm and 380 nm wavelengths. Ratios and sarcomere length transients were filtered using Savitzky–Golay filters. The parameters that are reported here for calcium transients are as follows: Fura signal amplitude, time to peak, time to 50% relaxation, and tau (time constant of exponential decay) (Fig.1). The parameters that are reported for sarcomere length profiles are as follows: diastolic length, shortening amplitude, time to peak shortening, and time to 50% relaxation time.

Sarcomere length records were time-shifted to allow for a fixed 15 ms delay introduced by the video analysis system. Data from individual myocytes were excluded from further analysis if their calcium transient or unloaded shortening profile was prolonged (>0.5 sec) or if calcium transients were not detected.

Some of the cells that had been stored in blebbistatin for 2 or 3 days were briefly exposed to blue light to test whether blebbistatin could be photoinactivated. These measurements were performed by measuring the function of several cells, exposing them to 460 nm light from a light emitting diode positioned 5 cm from the cells for 60 sec, and then repeating the functional measurements.

### Statistics

Statistical analyses were performed using the GLM function in SAS (SAS Institute Inc, Cary, NC). Post hoc comparisons against control values were performed using Dunnett's tests. Analyses of the effects of photoinactivation used a paired design. *P*-values less than 0.05 were considered significant. Values are reported as mean ± SEM.

## Results

### Myocyte morphology

**Figure 2:**
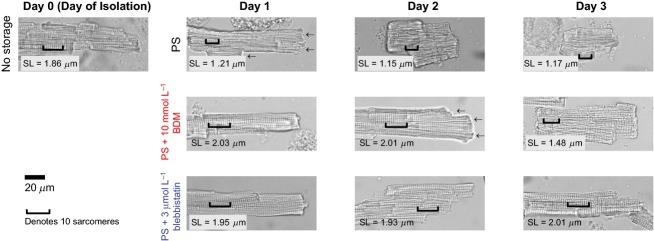
Cell morphology and sarcomere lengths. Top: Cells stored in perfusion solution (PS) show short sarcomere lengths (SL) and blebbing (arrows) after 1 day of storage at 4°C. Middle: Cells stored in PS + 10 mmol L^−1^ BDM maintain control sarcomere lengths for 2 days but begin to show blebbing. Cells shorten significantly after 3 days of storage. Bottom: Cells stored in PS + 3 *μ*mol L^−1^ blebbistatin maintain control sarcomere lengths and do not exhibit blebbing.

Freshly isolated myocytes that were tolerant to calcium had well-defined edges, clear striation patterns, and a distinctive rod-like shape (length to width ratio more than 2.5) (Fig.2).

**Figure 3:**
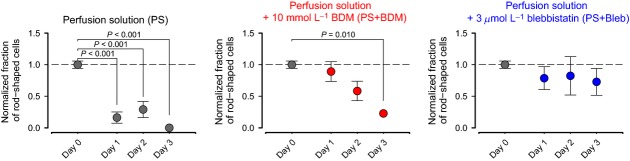
Rod-shaped cell counts plotted by days of storage for each of the three storage conditions.

Cells that were stored at 4°C without myosin ATPase inhibitors showed characteristic blebbing at their edges and had resting sarcomere lengths below 1.7 *μ*m after 24 h. The normalized fraction of rod-shaped cells was 0.16 ± 0.08 after 24 h (Fig.3; Supplemental Fig. S1 and S2) and zero within 3 days.

Cells that were stored in either 10 mmol L^−1^ BDM or 3 *μ*mol L^−1^ blebbistatin had sharper cell edges, more defined striation patterns, and were more likely to be rod-shaped (Fig.3) than cells stored without myosin ATPase inhibitors. Blebbistatin was more effective at maintaining these parameters than BDM. Sharp cell edges were still apparent in a batch of cells that were stored in blebbistatin at 4°C for 6 days (data not shown). Cells that were stored in BDM became significantly more ball-shaped or showed signs of short SL and blebbing by 72 h.

### Cell function

Calcium transients and unloaded shortening profiles for freshly isolated myocytes were similar to those measured in previous studies from our laboratory (Campbell et al. 2013; Chung and Campbell 2013). We were unable to record calcium transients or shortening profiles from cells that were stored for 24 h or more without myosin ATPase inhibitors.

**Figure 4:**
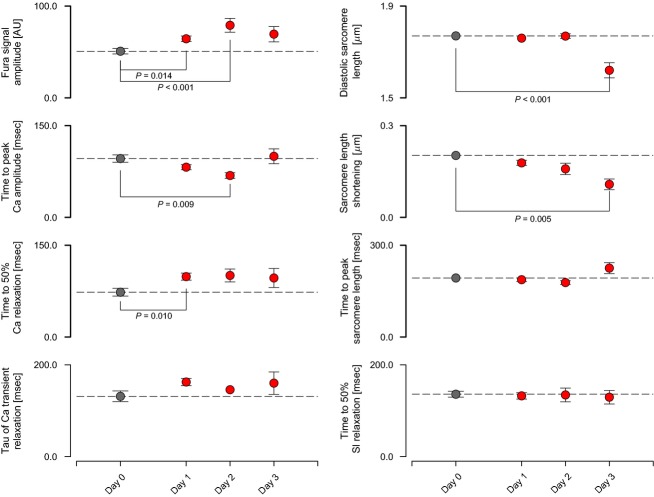
Effects of storage in 10 mmol L^−1^ BDM on calcium handling and contractile function.

Storage with 10 mmol L^−1^ BDM prolonged the viability of myocytes (PS + BDM, Fig.4). There were slight changes in the amplitude and timing of the Ca^2+^ transients after 24 h in BDM but contractile parameters (resting sarcomere length, shortening amplitude, time to peak shortening, and time to 50% relaxation) did not differ from control (Day 0) values after 2 days of storage. Resting sarcomere length and shortened amplitude both decreased after 3 days but the kinetics of the contraction remained unchanged.

**Figure 5:**
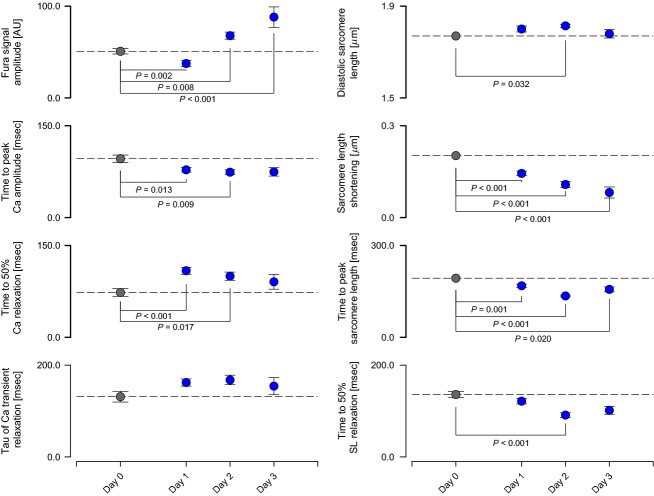
Effects of storage in 3 μmol L^−1^ blebbistatin on calcium handling and contractile function.

Storing cells in 3 *μ*mol L^−1^ blebbistatin was less effective at maintaining myocyte function than storing cells in BDM. As shown in Figure5, calcium transients were statistically different from control values after just 24 h of storage. The amplitude of shortening was also significantly reduced at this time-point and continued to decline in subsequent days.

**Figure 6:**
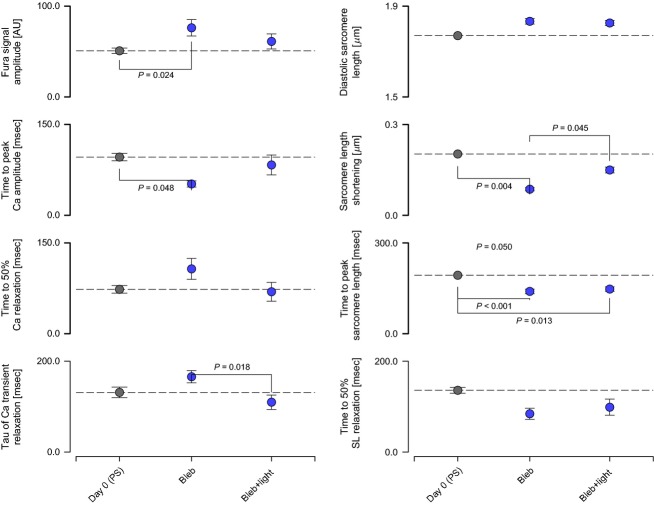
Effects of exposure to 460 nm light on calcium handling and contractile function in myocytes stored in 3 μmol L^−1^ blebbistatin.

Prior experiments have shown that blebbistatin can be photoinactivated by blue light (Sakamoto et al. 2005; Kabaeva et al. 2008). We studied 12 myocytes that had previously been stored in blebbistatin for 2 days before and after exposure to blue light to test whether light could be used to reverse the contractile inhibition. As shown in Fig.6, 60 sec of exposure to 460 nm light partially reversed some of the effects of blebbistatin on cell shortening and calcium.

## Discussion

This report shows that storage at 4°C with compounds that inhibit myosin ATPase can preserve the morphology and contractile function of isolated rat myocytes. Low temperature alone is insufficient. We did not test whether inhibiting myosin ATPase at room and physiological temperatures is sufficient to prolong function.

To our knowledge, our manuscript presents the first data quantifying contractile function and calcium handling in rat myocytes that have been stored for several days. Prior studies assessing storage options have focused on electrophysiology and used cells that were plated in cell culture and maintained at 37°C in incubators. This has some limitations. For example, cells typically lose their rod-like shape under normal culture conditions. Kabaeva et al. (2008) showed that this transition could be prevented using blebbistatin which is similar to our finding using noncultured cells. It is interesting to note that Kabeava et al. changed their bathing medium every 24 h while our protocol was much simpler. We left our cells in the refrigerator at 4°C until they were prepared for contractile assays. In the long run, our technique might be easier for most laboratories to implement.

Another complication of culturing cells is that the alignment of myofibers changes with the stiffness of the substrate that the cells are plated on (Hazeltine et al. 2012; Dasbiswas et al. 2015). These structural changes might alter contractile parameters and thus complicate the interpretation of experimental results.

### Cell function after storage using BDM

BDM maintained the functional viability of rat myocytes for 2 days. Parameters quantifying the unloaded sarcomere length shortening profiles were not statistically different from control values for at least 48 h (Fig.4). Only after 3 days of storage were diastolic sarcomere length and shortening amplitude attenuated. Calcium transients exhibited some statistically significant changes with storage time. For example, the time to 50% relaxation was prolonged after 24 h. The amplitude of the Fura signal also increased. Some of these effects may be related to changes in the way that cells load Fura after prolonged storage.

Although we did not specifically quantify cell membrane integrity using Trypan blue or similar staining techniques, we noticed a potential relationship between blebbing and cell viability. Rod-shaped cells with patent edges produced consistent, repeatable functional measurements. In contrast, myocytes that exhibited blebbing at their edges (Fig.2, Day 2 PS + BDM) more rapidly transitioned from their original rod-like shapes to amorphous balls when paced. Nonetheless, rod-shaped cells were viable in that they responded to the pacing stimulus even after several days of storage.

We limited the concentration of BDM to 10 mmol L^−1^ because it is just above the IC50 of force inhibition (Brixius et al. 2000) and typical of the conditions used to inhibit contractility in rodent myocytes (King et al. 2011; Campbell et al. 2013; and Chung and Campbell 2013). We did not test if increasing the concentration of BDM would improve cell viability. However, these experiments would need to be planned carefully because BDM's phosphatase activity might produce unforeseen effects (Wiggins et al. 1980).

In summary, storage in BDM at 4°C maintains the contractile properties of myocytes for 48 h after isolation. There are some minor changes in Ca^2+^ transients after 24 h but these may relate to changes in dye loading. The ability to use cells beyond the day of isolation could accelerate studies investigating the effects of small molecules on cardiac contractility (Malik et al. 2011).

### Cell function after storage using blebbistatin

Storing myocytes in solutions containing blebbistatin maintained cell morphology for several days (Fig.2) but altered calcium handling and contractile function (Fig.5). Time to peak calcium became shorter while Ca^2+^ relaxation was prolonged. Sarcomere length transients also became faster with storage but the most important functional difference was the progressive decline in shortening amplitude. The impaired shortening is broadly consistent with data reported for canine myocytes in a supplement published by Abi-Gerges et al. (2013).

It is interesting that storage in blebbistatin reduced contractility in these tests because the drug is membrane permeable and the solution changes that were performed before the contractile assays are calculated to reduce the drug's concentration below 3 nmol L^−1^. This is well below the IC50 for blebbistatin which is ∼2 *μ*mol L^−1^ (Limouze et al. 2004). Straight et al. (2003) have also shown that the effects of blebbistatin on cell cycling and cell division can be reduced by washout. In contrast, studies using cardiac cells suggest that the effects of blebbistatin are nearly irreversible by washout (Farman et al. 2008; Abi-Gerges et al. 2013). This suggests that blebbistatin dissociates from the myosin head at a very low rate.

We performed a simple test to investigate whether the inhibitory effects of blebbistatin on contraction could be relieved by modifying the compound with blue light (460 nm) (Kolega 2004; Sakamoto et al. 2005). As shown in Figure6, 60 sec of exposure partially reversed the inhibitory effects of blebbistatin. Increasing the exposure time, or using a light with a higher power, might enhance the reversal but could also cause cell damage and death (Kolega 2004; Sakamoto et al. 2005).

### Limitations

We performed qualitative assessments of cell morphology and quantitative assays of contractile function and calcium handling. We did not examine other cell structures, such as t tubule networks.

We also limited our study to rat myocytes because these are the preparations that our laboratory analyzes most frequently. Mouse myocytes are probably the most commonly used cell-type in cardiac studies and it would be interesting to see whether BDM and blebbistatin can prolong the viability of these preparations as well. It would also be useful to test whether BTS, the myosin ATPase inhibitor used by Abi-Gerges et al. (2013) in their study of canine myocytes, is effective in rodent cells as well. Because our study focused on the myosin inhibitors that are most commonly used with cardiac muscle (BDM and Blebbistatin) we did not perform this comparison.

### Summary

These data show that BDM can be used to maintain the morphology and prolong the contractile viability of rat cardiac myocytes for 48 h. Research groups may be able to leverage our findings and study cells for several days after they have been isolated. This will increase the efficiency and cost-effectiveness of experiments by reducing the number of isolations. It will also refine and reduce the use of animals in experiments investigating cardiac function (Russell and Burch 1959).

## Conflict of Interest

None declared.

## Supporting Information

**Figure S1.** Low magnification images showing rod- and nonrod-shaped cells. **Figure S2.** Cell survival counts.
