# Supplementary material for: Myocyte contractility can be maintained by storing cells with the myosin ATPase inhibitor 2,3 butanedione monoxime
Source: Physiol Rep. 2015 Jun 28;3(6):e12445. doi: 10.14814/phy2.12445 (PMC4522161; doi:10.14814/phy2.12445)
Supplement: Supplementary file 1 — Figure S1. Low magnification images showing rod- and nonrod-shaped cells. Figure S2. Cell survival counts. [file phy20003-e12445-sd1.pdf]

SUPPLEMENTARY FIGURES for
Myocyte contractility can be maintained by storing cells with the myosin ATPase inhibitor 2,3 butanedione monoxime

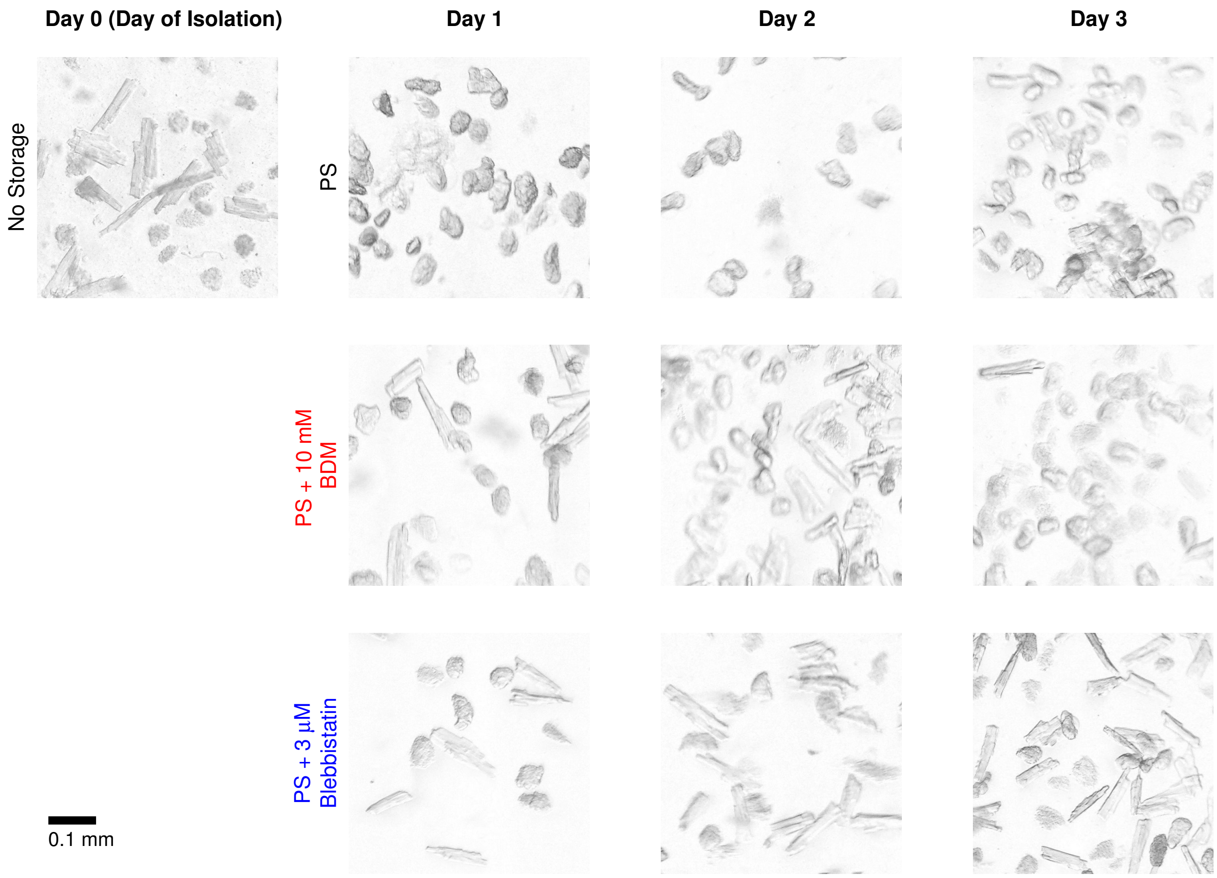

Supplementary Figure 1. Low magnification images showing rod- and non-rod-shaped cells. Variation in background intensity in these images was minimized using a rolling ball background subtraction. The brightness and contrast of the images were also enhanced.

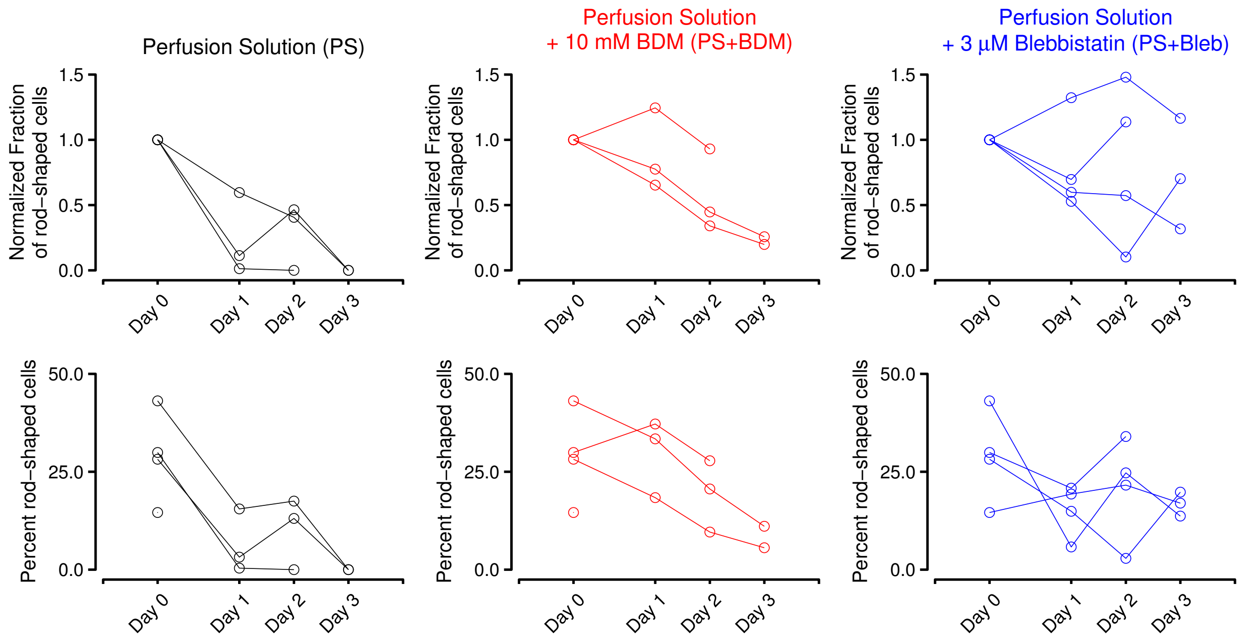

Supplementary Figure 2. Cell survival counts. Top row: Proportion of rod-shaped cells normalized to proportion on the day of isolation. Lines join symbols tracking individual isolations. Bottom row: As above, but showing the proportion of cells that are rod-shaped relative to the total number of rod-shaped and ball-shaped cells. Note that we did not perform centrifugation or purification of the isolate (Louch et al., 2011), so the proportions of cells that were rod-shaped upon isolation (Day 0) are lower than values that are sometimes reported in the literature.
